# The Role of the Toll-like Receptor 2 and the cGAS-STING Pathways in Breast Cancer: Friends or Foes?

**DOI:** 10.3390/ijms25010456

**Published:** 2023-12-29

**Authors:** Chiara Cossu, Antonino Di Lorenzo, Irene Fiorilla, Alberto Maria Todesco, Valentina Audrito, Laura Conti

**Affiliations:** 1 Department of Molecular Biotechnology and Health Sciences–Molecular Biotechnology Center “Guido Tarone”, University of Turin, Piazza Nizza 44, 10126 Turin, Italy; chiara.cossu@unito.it (C.C.); antonino.dilorenzo@unito.it (A.D.L.); 2 Department of Science and Technological Innovation (DISIT), University of Eastern Piedmont, 15121 Alessandria, Italy; irene.fiorilla@uniupo.it (I.F.); albertomaria.todesco@uniupo.it (A.M.T.); valentina.audrito@uniupo.it (V.A.)

**Keywords:** breast cancer, innate immunity, pattern recognition receptors, Toll-like receptor 2, cGAS/STING, chemoresistance

## Abstract

Breast cancer stands as a primary malignancy among women, ranking second in global cancer-related deaths. Despite treatment advancements, many patients progress to metastatic stages, posing a significant therapeutic challenge. Current therapies primarily target cancer cells, overlooking their intricate interactions with the tumor microenvironment (TME) that fuel progression and treatment resistance. Dysregulated innate immunity in breast cancer triggers chronic inflammation, fostering cancer development and therapy resistance. Innate immune pattern recognition receptors (PRRs) have emerged as crucial regulators of the immune response as well as of several immune-mediated or cancer cell-intrinsic mechanisms that either inhibit or promote tumor progression. In particular, several studies showed that the Toll-like receptor 2 (TLR2) and the cyclic GMP–AMP synthase (cGAS)–stimulator of interferon genes (STING) pathways play a central role in breast cancer progression. In this review, we present a comprehensive overview of the role of TLR2 and STING in breast cancer, and we explore the potential to target these PRRs for drug development. This information will significantly impact the scientific discussion on the use of PRR agonists or inhibitors in cancer therapy, opening up new and promising avenues for breast cancer treatment.

## 1. Introduction

Breast cancer is the most prevalent malignancy in women and ranks as the second leading cause of cancer-related deaths on a global scale [1]. Despite advancements in the treatment of breast cancer, a significant proportion of patients still advance to metastatic disease, posing a considerable challenge to effective therapy. The limited success of current therapies often stems from a predominant focus on targeting cancer cells alone, overlooking the intricate interactions within the tumor microenvironment (TME). Various cell populations in the TME engage in complex communication with cancer cells, facilitating cancer progression and resistance to treatments. In some instances, the complex crosstalk between cancer and immune cells leads to immunosuppression, redirecting the immune system toward a protumorigenic response.

Dysregulation of innate immunity is often associated with breast cancer and significantly contributes to inducing a chronic inflammatory state within the TME—a hallmark of cancer that contributes to all steps of cancer development and to resistance to current therapies [2]. Moreover, some of the receptors typically expressed by innate immune cells, such as pattern recognition receptors (PRRs), may promote tumor growth through intrinsic mechanisms within cancer cells [3].

**Figure 1:**
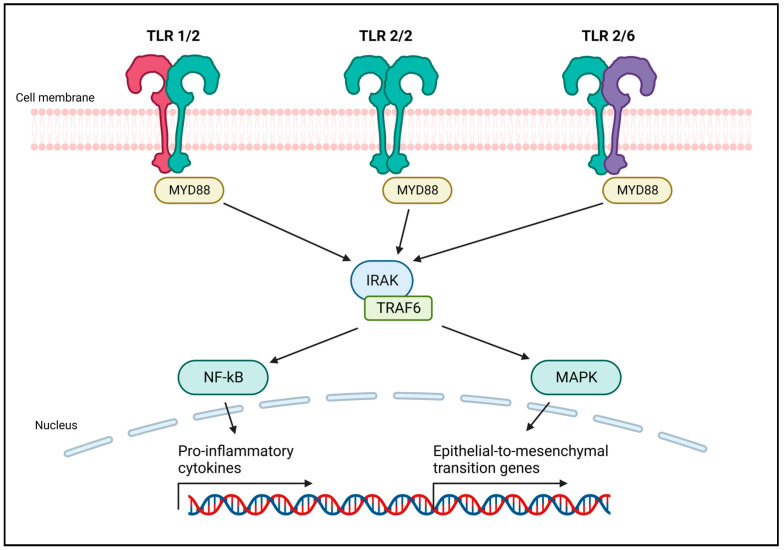
Schematic representation of TLR2 dimers and their signaling pathway. Like other TLRs, TLR2 forms homo- or heterodimers that allow for activation and signaling upon ligand binding. TLR2 islocalized in the outer cell membrane, and mainly dimerizes with TLR1 and TLR6. TLR2 uses the canonical MyD88 pathway to transduce a signal that, through the IRAK–TRAF6 complex, induces the activation of NF-κB and MAPK. NF-κB is responsible for the transcription of several pro-inflammatory cytokines. The MAPK pathway induces the epithelial to mesenchymal transition, promoting cancer cell invasion and metastasis. Created with BioRender.com (accessed on 22 December 2023).

Mounting evidence suggests a significant association between Toll-like receptors (TLRs) and the development of breast cancer [4,5]. TLRs exhibit a dual role in cancer: they can mediate tumor cell death, activating an effective antitumor immune response, or inhibit the immunosuppressive activity of myeloid-derived suppressor cells (MDSCs), preventing tumor growth [6,7]. However, they also possess protumorigenic functions [7]. This is particularly evident for TLR2, the most expressed TLR in triple negative breast cancer [4]. Indeed, data from the Kaplan–Meier plotter (kmplot.com (accessed on 21 December 2023)) and from the literature indicate that high expression of TLR2 is associated with poor relapse-free survival in breast cancer patients [8]. On the contrary, the expression levels of the other TLRs are not associated with poor prognosis in breast cancer (kmplot.com (accessed on 21 December 2023)). TLR2 activation by pathogen-associated or damage-associated molecular patterns (PAMPs or DAMPs, respectively), activates a signaling cascade initiated by Myeloid Differentiation Primary Response protein 88 (MyD88). The consequent recruitment of the interleukin-1 receptor-associated kinase (IRAK)–TNF Receptor Associated Factor (TRAF) complex leads to the activation of NF-κB and MAPK pathways. This induces the production of pro-inflammatory cytokines, and in cancer cells, may stimulate epithelial to mesenchymal transition (EMT) and proliferation (Figure 1). TLR2 promotes cancer cell survival and proliferation in breast [8] and gastric cancers [9], as well as in pancreatic ductal adenocarcinoma [10]. However, some studies suggested that TLR2 activation by PAMPs, or DAMPs such as high-mobility group box 1 (HMGB1) and heat shock proteins (HSPs), may promote anticancer immune responses [11,12,13]. The conflicting reports on TLR2’s antitumor and protumor properties underscore the critical need to comprehend its context-dependent role in breast cancer for potential therapeutic advancements.

Recent attention has focused on another PRR with a dual role in breast cancer, the stimulator of interferon genes (STING). The cyclic GMP–AMP synthase (cGAS) -STING pathway plays a crucial role in the innate immune system, recognizing cytosolic dsDNA [14]. Upon dsDNA binding, cGAS triggers cGAMP production, activating STING and the interferon regulatory factor 3 (IRF3) transcription factor. This cascade leads to the expression of type I interferons (IFNs), pro-inflammatory cytokines, and chemokines [15,16]. The consequent capability of the STING pathway to stimulate the cytotoxic activity of natural killer (NK) and cytotoxic CD8^+^ T cells [16] led to the development of many exogenous STING agonists used to stimulate anticancer immune responses. However, despite promising preclinical data, the majority of the clinical trials using STING agonists failed. One reason could lie in STING-induced activation of the NF-κB and MAPK pathways, which may foster cancer growth [17]. Indeed, chronic activation of STING may increase the expression of immunoregulatory genes [18] and promote breast cancer progression [19].

The emerging conflicting roles of TLR2 and STING in breast cancer progression, associated with their therapeutic potential, have prompted us to focus on the role of these two PRRs in breast cancer. Understanding the molecular mechanisms underlying their antitumor and protumorigenic effects holds promise for developing new combined therapies that could ameliorate cancer patients’ prognosis.

## 2. Role of TLR2 in Anticancer Immune Responses

TLRs are fundamental tools exploited by the immune system to trigger the innate immune response against pathogens and subsequently activate adaptive immunity. The immune surveillance is not only focused on preventing or fighting infections. It is also crucial for identifying and eliminating malignant cells that can initiate tumor development. When a tumor arises, the immune system engages in a complex process to counteract tumor progression, and attempts to resist cancer-induced immune suppression. In this context, the activation of TLRs by DAMPs released from cancer cells may enhance antigen presentation mechanisms in dendritic cells (DCs) and promote macrophage differentiation towards the M1 phenotype, known for its antitumor activity [20]. Consequently, TLR2 agonists have been evaluated in several solid tumor models as adjuvants for immune stimulation. For instance, the TLR2 natural ligand polysaccharide krestin (PSK) was able to stimulate TLR2 and elicit NK cell-mediated antitumor responses in different cancer models. In particular, PSK potentiated trastuzumab-induced antibody-dependent cell cytotoxicity of HER2^+^ breast cancer cells by stimulating NK cell activation [21]. To confirm that TLR2 stimulates NK cells, Ke et al. demonstrated that treatment with Strongylocentrotus nudus egg polysaccharide, a TLR2 agonist, induces NK cell proliferation, cytotoxicity and release of interleukin (IL)-2 and IFN-γ in a mouse model of lung cancer [22]. In lung cancer preclinical models, the administration of PAMPs or synthetic TLR2 ligands induced the differentiation of M1 macrophages that release nitric oxide, IFN-γ and pro-inflammatory cytokines, suggesting that TLR2 activation favors antitumor immune reactions [23]. In melanoma, TLR2 stimulation using the synthetic compound diprovocim was reported to induce antitumor activity in response to ovalbumin vaccination [24]. Similarly, in a fibrosarcoma study, combining vaccination against tumor-associated antigens with TLR2 agonists resulted in an increase in CD8^+^ T cells and antibody production, with a concomitant reduction in Treg frequency. However, the administration of TLR2 ligands alone produced the opposite effect, increasing Tregs. This suggests that TLR2 can yield both pro- and antitumoral effects, depending on the context [25].

Thus, the role of TLR2 in anticancer immune responses remains controversial and largely reliant on tumor types and models. TLR2 appears to possess the potential to either stimulate the immune system or suppress it. Part of this controversy might arise because TLR2 expression is not confined to the immune system. Many cancer cell types express TLR2 and take advantage on the activation of its signaling pathway, as will be discussed in the following paragraph.

## 3. The Protumoral Role of TLR2 in Breast Cancer

Immune cells exploit PRRs to detect disruptions in tissue homeostasis caused by cancer cells, thereby triggering an antitumor immune response. However, cancer cells can express PRRs as well, benefiting from the activation of their signaling pathways [26]. Among the PRRs, members of the TLR family exhibit an intriguing dual role in cancer. TLR2 mRNA expression is significantly higher in breast cancer than in normal tissues, with a higher expression in the triple-negative and HER2^+^ subtypes as compared to the luminal A and luminal B [8]. A significant increase in the levels of soluble TLR2 was observed in the sera from patients with both metastatic and non-metastatic breast cancer, as compared with healthy donors. Of note, soluble TLR2 was significantly higher in metastatic than in non-metastatic breast cancer patients, suggesting that TLR2 might be used as a biomarker to monitor disease progression [27]. Moreover, a significant correlation was observed between high TLR2 expression and poor prognosis in breast cancer patients [8]. Similarly, a positive correlation exists between the expression levels of its partners, TLR1 and TLR6, and the development of brain metastases in breast cancer patients [28], indicating the potential involvement of the TLR2 heterodimers in the metastatic spread of pre-existing tumors. Furthermore, high TLR2 mRNA levels are associated with poor relapse-free and overall survival in breast cancer patients who underwent surgery [8], as well as in patients treated with endocrine therapy or chemotherapy [8,29]. Importantly, TLR2 expression levels can predict the response to endocrine therapy with high accuracy in both luminal A and luminal B breast cancer patients [8]. Resistance to both endocrine therapy and chemotherapy has been associated with the presence of cancer stem cells (CSCs) [30]. TLR2 is expressed by breast cancer cells and promotes CSC self-renewal, invasiveness and drug resistance [31]. These effects result from various mechanisms, mostly dependent on the availability of TLR2 ligands, particularly DAMPs, in the TME. These molecules can be actively secreted by cancer cells or passively released during chemo- or radiotherapy, activating TLR2 signaling in either cancer or immune cells. Among the DAMPs that induce TLR2 activation, HMGB1 plays a major role. In the nucleus, HMGB1 is involved in the DNA repair processes and transcription, interacting with transcription factors like p53 [32]. However, HMGB1, as other moonlighting proteins, not only acts as a DNA-binding protein but also functions outside the cell. It can be actively secreted in response to cytokine stimulation or passively released from necrotic or damaged cells, subsequently inducing TLR2 activation [33]. Notably, breast cancer patients exhibiting high HMGB1 expression are more prone to develop cancer metastasis, especially in triple-negative breast cancer [34]. Other important DAMPs activating TLR2 include heat shock proteins (HSPs), a family of proteins that play a dual role. While their intracellular function involves supporting the correct folding or refolding of nascent and misfolded proteins, they can also be actively secreted or released by necrotic cells, binding to TLR2 in the TME. In breast cancer, the extracellular HSP90 co-chaperone Morgana acts as another significant activator of TLR2 [35], whereas versican has been identified as the DAMP involved in TLR2-mediated tumor promotion in glioma and lung cancer [36]. TLR2 serves also as a sensor of PAMPs, representing a bridge between eukaryotic cells and the microbial world, whose alterations may influence cancer development in different ways. In addition, TLR2 expressed on immune cells may not only exert antitumor activity but also lead to several immune suppressive effects that indirectly promote cancer progression [37].

Hence, we can classify TLR2’s protumoral effects into two primary categories: cancer cell-intrinsic and -extrinsic.

### 3.1. The Cancer Cell-Intrinsic Protumoral Effects of TLR2

In breast cancer cells, TLR2 is overexpressed and can be activated by endogenous ligands such as HSPs, HMGB1 and other DAMPs, or by exogenous ligands derived from pathogens, like bacterial lipoproteins. Once activated, TLR2 signaling promotes tumor growth and survival. Among the critical downstream molecules activated by TLR2 is NF-κB, which triggers the transcription of various pro-survival and pro-inflammatory genes [37]. Wenjie Xie and colleagues have demonstrated that TLR2 activation promotes breast cancer cell survival, proliferation and invasion through the activation of NF-κB and the secretion of protumoral cytokines. They also reported a 10-fold higher TLR2 expression in the invasive MDA-MB-231 human triple-negative breast cancer cells compared to the poorly invasive ER^+^ MCF-7 cells [38]. TLR2 plays a role in the regulation of CSCs, a subpopulation of tumor cells with self-renewal and tumor-initiating capabilities. Activation of TLR2 in mammary epithelial stem cells and in breast cancer cells enhances the expression of stemness-related genes, promoting the acquisition of CSC properties that contribute to treatment resistance and tumor recurrence [39]. We have previously demonstrated the upregulation of TLR2 in breast CSC-enriched tumorspheres compared to epithelial-like breast cancer cells. We showed that the HMGB1-TLR2-NF-κB axis promotes CSC self-renewal through the release of IL-6 and tumor growth factor-β (TGF-β), subsequently activating STAT3 and Smad3 [31]. More recently, we demonstrated that TLR2 promotes breast cancer progression and metastasis in HER2^+^ transgenic mice in a cancer cell-intrinsic way. Indeed, tumor growth was impaired in HER2^+^ TLR2^KO^ mice as compared to their TLR2^WT^ littermates. Transplantation experiments using breast cancer cell lines derived from these mice demonstrated that TLR2^KO^ cells were not tumorigenic, in contrast to TLR2^WT^ cells. However, as discussed in the next section, TLR2 also contributes to tumor progression in a tumor cell-extrinsic manner, by shaping an immunosuppressive TME [29]. Similarly, TLR2 deletion impairs tumorigenesis in MMTV–Wnt1 transgenic mice that spontaneously develop ER^neg^ mammary tumors. Of note, TLR2 further upregulates the transcription of Wnt-dependent genes (including Cd44 and Lgr5) [39], which serve as significant mediators of NF-κB protumorigenic activity [40].

**Figure 2:**
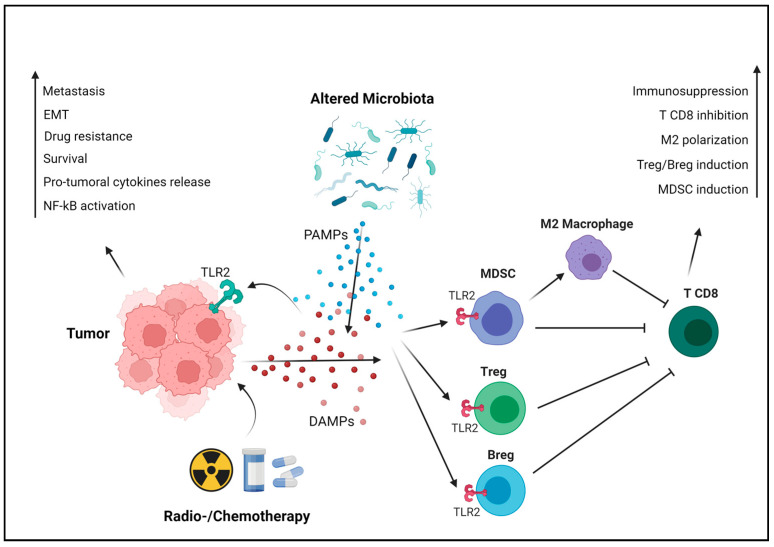
TLR2-mediated protumoral mechanisms. Cancer cells actively or passively release DAMPs, especially following cell death induced by chemo- or radiotherapy. In addition, alterations in the microbiota due to antibiotic therapies, chemotherapies, or other factors can foster the proliferation of bacterial species that exert protumoral activity, releasing PAMPs in the TME. These endogenous and exogenous TLR2 ligands activate its signaling, promoting tumor progression in a cancer cell-intrinsic manner (on the left). TLR2 signaling activates NF-κB, which subsequently transcribes protumoral cytokines such as IL-6, TGF-β and VEGF. This stimulates cancer cell survival, angiogenesis and resistance to therapies. Additionally, TLR2 triggers the MAPK pathway, inducing EMT and promoting metastasis. Moreover, TLR2 is expressed by immune cells, where it exerts immunosuppressive effects favoring tumor progression in a cancer cell-extrinsic manner (on the right). TLR2 induces the differentiation of T and B regulatory cells, as well as of MDSCs from bone marrow precursors. MDSCs contribute to immunosuppression through several mechanisms, including the reprogramming of macrophages into the M2 phenotype. These processes collectively result in the production of cytokines that inhibit CD8^+^ T cells and their activity, promoting tumor immune evasion. Created with BioRender.com (accessed on 10 November 2023).

Apart from its direct effect on tumorigenesis, we have demonstrated that TLR2 mediates breast cancer resistance to doxorubicin and other drugs, inducing immunogenic cell death (ICD) and the release of DAMPs that activate TLR2 signaling in cancer cells, thereby enhancing their survival [29]. In addition to NF-κB, TLR2 signaling activates the MAPK pathway, involved in cell proliferation and migration. In gastric cancer, this activation leads to the EMT, a pivotal process linked to cancer cell invasion and metastasis. TLR2-mediated EMT allows cancer cells to acquire a more migratory and invasive phenotype, facilitating their dissemination to distant sites and the formation of metastasis (Figure 2) [41]. Another important aspect of TLR2’s protumoral role is its impact on angiogenesis, the process of forming new blood vessels to supply nutrients and oxygen to growing tumors. TLR2 activation in breast cancer cells upregulates the expression of vascular endothelial growth factor (VEGF) and other pro-angiogenic factors, fostering the formation of new blood vessels that support tumor growth [38].

Beyond its direct effects on cancer cells, TLR2 may also confer protection from CD8^+^ T cell killing. Other TLRs, such as TLR4, induce the expression of immune checkpoint molecules like programmed death-ligand 1 (PD-L1), which can lead to T-cell exhaustion and suppress antitumor immune responses [42]. However, the correlation between TLR2 and PD-L1 expression requires further elucidation.

TLR2 serves as both a DAMP detector and a sensor of PAMPs, connecting TLR2-expressing cells with the microbial world. This mechanism has been largely studied in the immune system for its protective role against infections. Recently, researchers have focused on studying the role of the microbiota in different diseases. Alterations in the microbiota composition, and its interaction with our cells, might be involved in many pathogenic processes, including carcinogenesis and cancer recurrence [37,43]. Several studies have demonstrated the correlation between specific bacterial species and tumor development, especially in the gastrointestinal system. For instance, Helicobacter pylori contributes to gastric and colon carcinogenesis through various mechanisms, including TLR2-mediated activation of NF-κB and Wnt, and the induction of EMT in epithelial cells [44,45].

A specific microbiota has been detected in the mammary gland, altered in breast cancer. Bacteroides fragilis is found in tumor biopsies of breast cancer patients, and promotes tumorigenesis. Fusobacterium nucleatum (*F. nucleatum*), an oral commensal bacterium, can spread to other organs and become pathogenic. Indeed, *F. nucleatum* is detected in colon and breast cancer tissues, directly promoting tumor progression by activating TLR2 signaling in cancer cells and inducing immunosuppression, as described in the following paragraph [46,47,48].

Beyond the individual protumoral role played by specific bacterial species under certain conditions, it is important to note more complex alterations in the microbiota across various parts of the body. These conditions, called dysbiosis, can cause carcinogenesis or promote the progression of existing tumors by enhancing resistance to therapies and the spread of metastasis. Correlations between antibiotic therapies before cancer diagnosis or in its early stages and poorer prognosis in breast cancer patients have been reported. This is probably caused by the establishment of dysbiosis and increased presence of protumoral bacteria. Furthermore, chemotherapy can induce dysbiosis and opportunistic infections, increasing the availability of TLR2 ligands and potentially limiting therapy effectiveness [49,50,51].

Collectively, this evidence strongly suggests that in breast cancer, the presence of TLR2 along with its endogenous or exogenous ligands can significantly influence a worse prognosis.

### 3.2. The Cancer Cell-Extrinsic Protumoral Effects of TLR2

TLR2 does not just impact cancer cells directly; it also plays a significant role in cancer progression through its influence on the immune system. When activated, TLR2 can either promote or hinder tumor growth, depending on the specific immune cells involved. Tregs, MDSCs, neutrophils and macrophages express TLR2, and upon its activation, they contribute to cancer progression and metastasis due to their immunosuppressive functions [37]. Treg frequency is significantly decreased both in the TME and in the periphery in TLR^KO^ breast tumor-bearing mice, since TLR2 activation on Treg cells induces their expansion [29,52]. Moreover, upon TLR2 stimulation, macrophages release chemokines that recruit Treg [53]. Tregs subsequently inhibit the antitumor activity CD8^+^ T cells through the release of immunosuppressive cytokines, like IL-10 [37]. Similarly, TLR2 activation in B lymphocytes induces their differentiation into regulatory B cells (Bregs) that produce IL-10 and suppress the T-cell antitumor response [54]. TLR2 activation in CD4^+^ T cells, triggered by HSP90 on autophagosomes released by breast cancer cells, initiates an autocrine IL-6 cascade. This process induces the expression of IL-10 and IL-21, fostering immune suppression and inhibiting antitumor responses [55]. TLR2 activation in bone marrow precursors induces their differentiation to MDSCs, which accumulate in the TME and release protumoral cytokines. Moreover, this contributes to the polarization of macrophages towards the protumoral M2 phenotype and the release of nitric oxide (NO), a potent inhibitor of effector T cells. DAMPs such as HMGB1 and serum amyloid A 1 protein, secreted by breast cancer cells and elevated in the plasma and tumor biopsies from patients with advanced triple-negative breast tumors, induce an immunosuppressive response in neutrophils via TLR2 [34,56]. Specifically, HMGB1 prompts the release of neutrophils’ extracellular traps, favoring the development of lung metastasis in triple-negative breast cancer mouse models [34].

These mechanisms collectively create an immunosuppressive microenvironment that supports tumor progression. TLR2 activation within immune cells triggers the release of various factors that hinder immune responses against tumors, promoting their growth and spread. Understanding these interactions helps identify potential targets to disrupt the immunosuppressive TME and bolster antitumor immunity.

## 4. Role of cGAS-STING in Antitumor Immunity

Besides its crucial functions in the immune response against pathogens, the cyclic GMP-AMP synthase (cGAS)-stimulator of interferon genes (STING) pathway plays pivotal role in the antitumoral immune response. This pathway is widely expressed in immune cells as well as cancer cells, influencing carcinogenesis through various mechanisms. One such mechanism involves the induction of a robust type I interferon (IFN) response mediated by cGAS-STING activation by tumor-derived DNA in immune cells and tumor cells themselves [57]. Type I IFNs enhance the tumor immunogenicity and facilitates the adaptive immune response against cancer cells [58]. In the context of cancer, the cGAS-STING pathway is activated by cytosolic DNA derived from chromosomal instability (CIN), a hallmark of cancer [59,60], or by DNA damage resulting from cancer therapy. Upon activation in tumor cells, the release of type I IFNs, characteristic of “hot” tumors, shapes the TME and triggers an antitumor response. Type I IFNs target DCs in the TME, which play a crucial role in initiating tumor specific-T cell responses and eliminating the tumor [61]. The activation of the cGAS-STING pathway can also trigger downstream NF-κB signaling, which plays a significant role in regulating tumor growth. Under certain circumstances, the activation of the canonical NF-κB pathway may synergize with cGAS-STING activation, enhancing the type I IFN response and strengthening the antitumor immune defense [62]. In addition to cell-intrinsic DNA sensing in tumor cells, the cGAS-STING pathway can be activated in immune cells within the TME by tumor-derived DNA via membranous vesicles, such as exosomes [63], which fuse with immune cell membrane. DCs take up extracellular DNA, resulting in a type I IFN response that, in turn, increases tumor-infiltrating DCs and enhances presentation to CD8^+^ T cells [64]. Furthermore, other studies have demonstrated that not only tumor-derived DNA but also the immunostimulatory second messenger cGAMP released by tumor cells can activate the cGAS-STING pathway [65,66]. Tumor-derived cGAMP can be transferred to immune cells in the TME, triggering the release of type I IFNs via STING, which, in turn, activates NK cells, crucial for antitumor immunity [67,68]. Additionally, the uptake of cGAMP has been observed also in monocytes and macrophages [69], where it activates STING signaling and leads to reprogramming of M2 tumor-promoting macrophages to a M1 antitumor phenotype [70]. However, a pan-cancer analysis has demonstrated that elevated activation of the cGAS-STING pathway, particularly of IRF3, is associated with poor prognosis in patients diagnosed with specific types of cancer, including colorectal, prostate and lung adenocarcinomas [71]. Indeed, it is becoming more and more evident that the cGAS-STING pathway exerts a controversial role in cancer, supporting diverse and sometimes opposing functions, favoring tumor progression in some contexts. These aspects will be dissected in the following paragraph.

## 5. The Protumoral Role of cGAS-STING in Breast Cancer

As discussed above, the cGAS-STING pathway plays an important role in antitumor adaptive immunity, involving the release of type I IFNs that shape the TME in an antitumoral setting. Consistently, defective STING signaling has been suggested to promote tumorigenesis and host immunosurveillance evasion [72]. Various studies have demonstrated that epigenetic silencing of cGAS and STING genes, rather than mutation, occurs in different types of cancer [73,74]. However, cGAS-STING signaling has also been linked to cancer cell survival and tumor progression. In the context of breast cancer, it has been shown that cGAS-STING signaling activation often yields paradoxical outcomes. While the type I IFN response induced by acute STING activation has antitumor effects, persistent activation of this pathway results in chronic inflammation. This induces a shift from type I IFN response and canonical NF-κB signaling to noncanonical NF-κB signaling, contributing to tumor-promoting effects [75,76]. Persistent activation of cGAS-STING signaling has been observed in different tumor types with high levels of CIN, including breast cancer, resulting in tumor progression, invasion, and metastasis formation [75,77] through both cell-intrinsic and cell extrinsic mechanisms.

**Figure 3:**
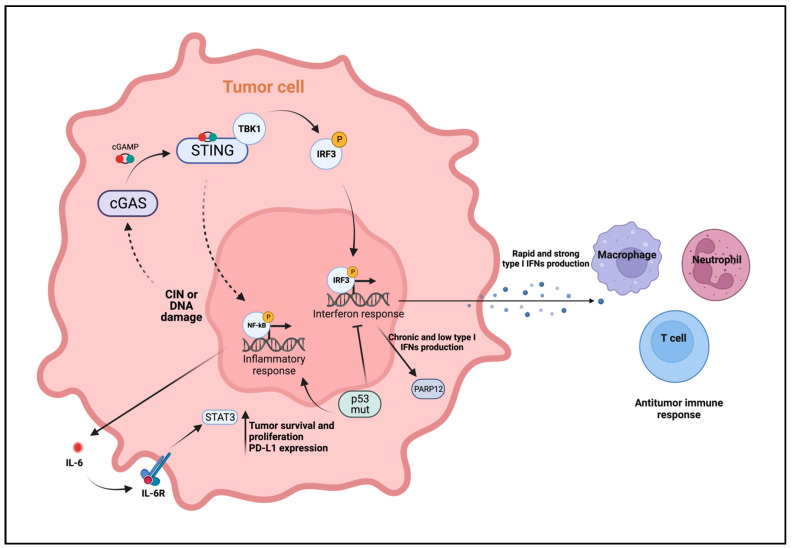
cGAS-STING antitumoral and protumoral mechanisms. This graphical representation illustrates the antitumoral and protumoral roles played by the cGAS-STING signaling pathway. In tumor cells, the activation of the cGAS-STING pathway is predominantly triggered by CIN or DNA damage induced by chemo- and radiotherapy. Upon activation, double-stranded DNA fragments bind to cGAS, leading to the synthesis of cGAMP. Subsequently, cGAMP binds to STING dimers in the endoplasmic reticulum (ER) membrane, activating STING and causing its trafficking to an ER-Golgi intermediate compartment. In this compartment, TBK1 is recruited to phosphorylate STING, and this phosphorylation, in turn, recruits IRF3. Phosphorylated IRF3 can form dimers, translocate to the nucleus, and activate the transcription of target genes, including type I IFNs, that are released by tumor cells, recruiting antitumor immune cell populations. Conversely, a chronically low production of type I IFNs activates PARP12, whose overexpression is associated with poor prognosis in breast cancer patients. Furthermore, dysregulated cGAS-STING signaling can activate the transcription factor NF-κB, leading to chronic inflammation and tumor-promoting effects. This includes the activation of the IL-6-STAT3 axis, promoting pro-survival effects, tumor cell proliferation, and PD-L1 expression, resulting in immune escape. Mutated forms of p53 play a role in the transition from the canonical cGAS-STING-TBK1-IRF3 signaling to the cGAS-STING-NF-κB signaling, contributing to the tumor-promoting effects of the cGAS-STING pathway. Created with BioRender.com (accessed on 14 November 2023).

In the context of breast cancer, CIN and the subsequent DNA damage occur frequently [77] and are known to activate cGAS-STING signaling. It has recently been reported that tumor cells with CIN rely on the cGAS-STING pathway to promote cancer cell survival through the STING-mediated NF-κB activation, which in turn induces the expression of IL-6 and the subsequent activation of STAT3 [78]. Furthermore, DNA damage induced by chemotherapy in triple negative breast cancer cells triggers the cGAS-STING pathway, leading to NF-κB activation and the pro-survival IL-6-STAT3 axis. This promotes immune escape by upregulating PD-L1 expression [79]. Additional studies reveled that PD-L1 expression by breast cancer cells contributes to the expression of the IFN-related DNA damage resistance signature (IRDS), a subset of IFN-induced genes that protect cancer cells from DNA damage. This mechanism is sustained by low and persistent levels of type I IFNs due to the DNA damage-induced cGAS-STING activation [80]. Similarly, another study highlights that genotoxic stress induced by chemotherapy sustains DNA damage response and breast cancer cell resistance by triggering chronic cGAS-STING-dependent IFN production. This, in turn, induces the expression of PARP12, a member of the ADP-ribosyl transferases family involved in the control of protein translation and inflammation, whose high expression is associated to poor prognosis in breast cancer patients [81]. Conversely, conflicting studies stated that chronic activation of cGAS-STING signaling by CIN or DNA damage induced by radio- and chemotherapy results in the downregulation of type I IFN response. Instead, it promotes downstream alternative NF-κB signaling, leading to the upregulation of EMT-related gene expression, supporting tumor invasion and metastasization [75,82]. In addition, mutated p53, but not wild-type p53, has been reported to suppress the canonical cGAS-STING-TBK1-IRF3 axis in breast cancer. This occurs by binding to TBK1 and preventing the formation of the trimeric STING-IRF3-TBK1 complex [83]. Ultimately, this results in reduced type I IFN production, switching to the alternative NF-κB signaling (Figure 3). Collectively, these studies indicate that breast tumor cells can rewire the cGAS-STING signaling to promote cancer cell survival, tumor progression and metastasization.

## 6. Therapeutic Potential of Innate Immune Molecules Targeting for Breast Cancer Treatment

### 6.1. Activation

Innate immunity serves as our primary defence against various diseases, including cancer, and acts as a vital intermediary for the activation of adaptive immunity. Consequently, employing agonists capable of stimulating innate immune pathways appears to be a logical approach in the realm of anticancer therapies. Despite the considerable attention these molecules have garnered in the past decade among researchers, they remain relatively unexplored as therapeutic targets. Particularly in breast cancer, only a limited number of studies have been published on this subject.

To date, the majority of the investigations on TLR2 ligands have centered on Pam3CSK4. This agonist has been utilized to enhance vaccination efficacy by augmenting antigen presentation by DCs and, consequently, CD8^+^ T cell activation in preclinical cancer therapy [84,85,86]. Recently, Wei Shi and colleagues demonstrated the effectiveness of a self-assembled vaccine targeting tumor-specific antigens combined with TLR2 agonists as an immunotherapeutic approach against breast cancer. This combination was found to induce DC maturation and bolster the CD8^+^ T cell response [87]. Additionally, a study by Bichern Liu and colleagues demonstrated that administering Streptococcus-derived PepO protein in a triple negative breast cancer mouse model stimulates antitumor immunity by steering macrophage differentiation towards the M1 phenotype through TLR2 signaling [88]. Prior research by Hailing Lu and colleagues showed that the mushroom-derived polysaccharide krestin (PSK) exhibits antitumor activity by triggering an immune response in NK cells via TLR2 activation. Hence, PSK synergizes with trastuzumab, enhancing its ability to mediate antibody-dependent cell cytotoxicity (ADCC) in a mouse model of HER2^+^ mammary cancer. Similar results were obtained using human NK cells and MDA-MB-231 cells [21,89]. However, apart from these studies, there is a lack of other preclinical studies on TLR2 activation as a treatment for breast cancer. According to our knowledge, clinical trials administering TLR2 agonists in breast cancer patients have not been conducted.

**Figure 4:**
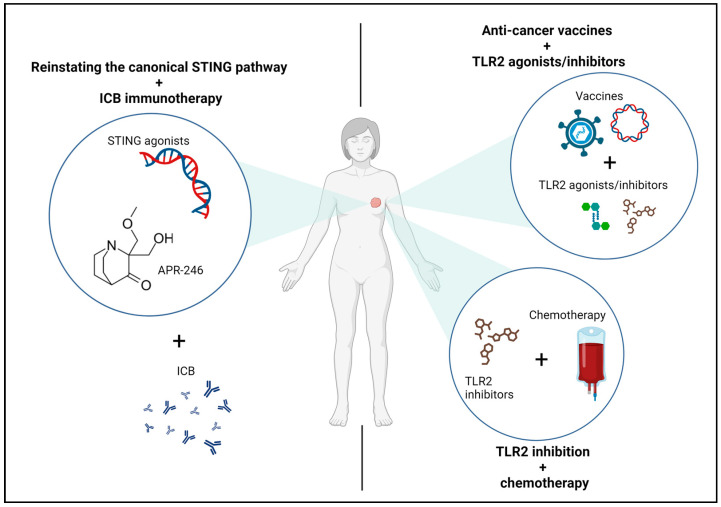
Approaches to PRR-targeted therapies for breast cancer. Schematic representation of the therapeutic approaches proposed in this review. Left panel: The cGAS-STING pathway exhibits alterations in various tumors, including breast cancer. The pathological shift of this signaling, from an immunostimulatory state to a protumoral one, can be reversed by utilizing STING agonists combined with agents that restore canonical NF-κB and IRF3 pathways, hindering cancer progression (left circle). The proposed combination of STING agonists with the drug APR-246, known for reactivating the tumor suppressor P53 in P53-mutated tumors, is suggested here. The efficacy of these therapies could potentially be enhanced through combination with ICB immunotherapies, such as anti-PD-L1 antibodies. Right panel: Conflicting data exist in the current literature concerning the role of TLR2 in cancer. Consequently, the use of agonists or inhibitors must be carefully evaluated based on the specific context. TLR2 agonists appear promising in enhancing antigen presentation when coupled with anticancer vaccines (right upper circle). However, considering the protumoral roles of TLR2 as described in this review, TLR2 inhibition might serve as an alternative strategy, particularly in tumor subtypes where TLR2 correlates with poor prognosis. Furthermore, TLR2 inhibition has shown promising outcomes when combined with chemotherapies inducing immunogenic cell death, thereby releasing DAMPs capable of activating TLR2 signaling and its described protumoral effects. Created with BioRender.com (accessed on 14 November 2023).

In contrast, the stimulation of cGAS-STING signaling is a strategy that has shown promising results in different tumors in preclinical models. The study of STING agonists has gained ground in recent years, either as standalone treatment or in combination with other therapies. Recently, Corrales at al. demonstrated that the intratumoral injection of STING agonists leads to an effective antitumor T-cell response triggered by a potent type I IFN production [90]. Furthermore, STING agonist cyclic dinucleotides can serve as potent adjuvants for radiotherapy, enhancing the adaptive antitumor response and reducing the immune suppressive microenvironment by reprogramming M2 macrophages [91]. Concerning breast cancer, different STING agonists have been employed to enhance the antitumor response, mostly in combination with other strategies. Systemic administration of cGAMP, the endogenous ligand of STING, showed high efficacy in suppressing tumor growth and reducing lung metastasis in a mouse model of triple negative breast cancer [92]. Other STING agonists, such as the c-di-GMP, have been shown to increase the potency of a bacterial-based vaccine for metastatic breast cancer in the mouse triple negative breast cancer 4T1 model. This approach suppresses metastasization and impairs tumor growth by targeting MDSCs [93]. Due to the high expression of PD-L1 in many types of breast cancer, STING agonists have been tested in combination with immune checkpoint blockades (ICB). It has been observed that STING agonists combined with atezolizumab have a synergistic effect in improving the antitumor response in a model of breast cancer by increasing CD8^+^ T cells and reducing Tregs infiltrating the tumor [94]. cGAS-STING exogenous activation can also promote NK cell antitumor immunity. Lu et al. demonstrated that treatment with high doses of cGAMP can prime the signaling activation in NK cells from PMBCs of patients with cancer [68]. Conversely, another study indicates that direct uptake of cGAMP by NK cells results in cell death, while the delivery of encapsulated STING agonists is able to indirectly activate NK cells by STING signaling activation in DCs [95]. As discussed in the previous paragraphs, mutated forms of p53 can interfere with antitumor activities of the cGAS-STING signaling in breast cancer, while wild-type p53 contributes to its activation [83]. For this reason, pharmacological reactivation of p53 could be an effective strategy to reactivate the cGAS-STING-dependent IRF3 signaling in tumor cells, promoting cancer cell apoptosis and modulating the TME [83] (Figure 4).

Despite the promising outcomes observed in preclinical tumor models, the translation of STING agonists into successful clinical trials has proven challenging. This discrepancy can be attributed to the limited efficacy of different STING agonists, such as the 5,6-dimethylxanthenone-4-acetic acid (DMXAA), which was the inaugural STING agonist evaluated in clinical trials and the sole one to reach phase III. These agonists have demonstrated efficacy restricted to murine STING [96]. Notably, structural analyses of human and murine STING underscored the significance of developing STING agonist derivatives specifically binding to the human receptor [97]. Furthermore, the inherent instability of the majority of STING agonists in vivo has hindered their therapeutic effectiveness. Consequently, numerous preclinical trials across various solid cancers are underway to explore formulations aimed at circumventing this challenge. Strategies include incorporating these molecules into nanoparticles or inducing their in vivo production by bacteria or cells. However, as of now, no reported outcomes exist regarding breast cancer patients’ responses to these trials [98].

### 6.2. Inhibition

Considering TLR2’s multifaceted role in fostering protumoral and immunosuppressive effects, as delineated in preceding sections, we propose an innovative therapeutic strategy centered on TLR2 inhibition (see Figure 3). The ambiguous nature of TLR2 in cancer necessitates a meticulous approach, weighing the use of agonists or inhibitors contingent upon specific contexts. While TLR2 stands as a crucial molecule for the immune system, insights from TLR2^KO^ mice, exhibiting no evident alterations in immune cells or other functions, support the feasibility and safety of its inhibition [29,99]. However, no clinical trials have been conducted, or are currently ongoing, pertaining to TLR2 inhibition in breast cancer or other solid tumors. Nevertheless, the utilization of TLR2-targeting monoclonal antibodies or inhibitors has showcased promise in hematological malignancies clinical trials [100]. Studies in mouse models of various cancers, such as gastric and pancreatic cancer, have revealed that genetic deletion of TLR2 or inhibition using blocking antibodies like OPN-301 hindered tumorigenesis [41]. Recent publications have also highlighted the impact of natural compounds like Robinin in inhibiting pancreatic cancer progression through the modulation of the TLR2-PI3k-Akt signaling pathway [101]. Moreover, investigations in head and neck squamous cell carcinoma demonstrated that TLR2 blocking antibodies restrained the growth of organoids and patient-derived xenografts (PDXs) [102]. Despite promising outcomes observed with TLR2 antagonistic targeting in various cancer models, there remains a lack of investigations into TLR2 inhibition as a treatment for breast cancer. Recently, our team published a study demonstrating that TLR2 promotes breast cancer progression in preclinical models, both through cancer cell-intrinsic mechanisms and immunosuppression. Notably, TLR2 plays a pivotal role in chemotherapeutic resistance since chemotherapy triggers the release of DAMPs, activating its signaling pathway. Importantly, inhibition of TLR2 using the small molecule CU-CPT22 potentiated the efficacy of chemotherapy both in vitro and in vivo [29]. We believe that our study, coupled with existing data on TLR2 targeting in diverse cancers, could pave the way for a novel approach in breast cancer treatment, warranting comprehensive exploration in the forthcoming years.

## 7. Conclusions

The wide array of PRRs exhibiting both tumor-suppressive and tumor-promoting functions presents an unparalleled prospect for the development of anticancer treatments aimed at either enhancing or inhibiting their signaling pathways. However, a comprehensive understanding of how a particular receptor operates in a specific cancer environment is crucial to avoid inadvertently bolstering tumor-promoting immune suppression or instigating prolonged inflammation during treatment. In the context of breast cancer, TLR2 and STING emerge prominently among PRRs for their potential in pioneering new therapeutic approaches. Modulating these specific PRRs holds the promise of triggering both intrinsic anticancer effects within tumor cells and eliciting immune-mediated anticancer responses. Such modulation may augment the sensitivity of cancer cells to chemotherapy and ICB. Directly inhibiting TLR2 with antagonists or addressing elements within the gut or tumor microbiota that trigger its activation using antimicrobial agents could potentially heighten the efficacy of certain anticancer treatments in tumors resistant to standard therapies. Simultaneously, activating the cGAS-STING pathway in immune cells could foster T lymphocyte recruitment within the TME and restore an antitumor immune microenvironment. It is worth noting that potential challenges associated with employing drugs that modulate these PRRs in cancer immunotherapy could be mitigated by targeting these drugs specifically to breast cancer or immune cells, utilizing selectively targeted nanoparticles or other sophisticated delivery systems. The integration of these strategies with chemotherapy and/or ICB paves the way for the development of innovative therapies in the ongoing battle against breast cancer.

## Data Availability

No new data were created or analyzed in this study. Data sharing is not applicable to this article.

## References

[1] Siegel R.L., Miller K.D., Wagle N.S., Jemal A. (2023). Cancer Statistics, 2023. CA A Cancer J. Clin..

[2] Salemme V., Centonze G., Cavallo F., Defilippi P., Conti L., Albini A., Ferrone S., Bruno A., Lollini P.-L. (2021). The Crosstalk Between Tumor Cells and the Immune Microenvironment in Breast Cancer: Implications for Immunotherapy. Front. Oncol..

[3] Man S.M., Jenkins B.J. (2022). Context-Dependent Functions of Pattern Recognition Receptors in Cancer. Nat. Rev. Cancer.

[4] Shi S., Xu C., Fang X., Zhang Y., Li H., Wen W., Yang G. (2020). Expression Profile of Toll-like Receptors in Human Breast Cancer. Mol. Med. Rep..

[5] Chandrasekar S.A., Palaniyandi T., Parthasarathy U., Surendran H., Viswanathan S., Wahab M.R.A., Baskar G., Natarajan S., Ranjan K. (2023). Implications of Toll-like Receptors (TLRs) and Their Signaling Mechanisms in Human Cancers. Pathol. Res. Pr..

[6] Wang J., Zhang J., Wang J., Hu X., Ouyang L., Wang Y. (2023). Small-Molecule Modulators Targeting Toll-like Receptors for Potential Anticancer Therapeutics. J. Med. Chem..

[7] Dajon M., Iribarren K., Cremer I. (2017). Toll-like Receptor Stimulation in Cancer: A pro-and Anti-Tumor Double-Edged Sword. Immunobiology.

[8] Turnbull A., Gerratana L., Matteo Luca Battisti N., Marsden R., Yang J., Wang Y., Liu S., Zhang Y. (2020). Dysregulation of TLR2 Serves as a Prognostic Biomarker in Breast Cancer and Predicts Resistance to Endocrine Therapy in the Luminal B Subtype. Front. Oncol..

[9] West A.C., Tang K., Tye H., Yu L., Deng N., Najdovska M., Lin S.J., Balic J.J., Okochi-Takada E., Mcguirk P. (2017). Identification of a TLR2-Regulated Gene Signature Associated with Tumor Cell Growth in Gastric Cancer. Oncogene.

[10] Lundy J., Gearing L.J., Gao H., West A.C., Mcleod L., Deswaerte V., Yu L., Porazinski S., Pajic M., Hertzog P.J. (1992). TLR2 Activation Promotes Tumour Growth and Associates with Patient Survival and Chemotherapy Response in Pancreatic Ductal Adenocarcinoma. Oncogene.

[11] Takeuchi O., Akira S. (2010). Leading Edge Review Pattern Recognition Receptors and Inflammation. Cell.

[12] Gardai S.J., Mcphillips K.A., Frasch S.C., Janssen W.J., Starefeldt A., Murphy-Ullrich J.E., Bratton D.L., Oldenborg P.-A., Michalak M., Henson P.M. (2005). Cell-Surface Calreticulin Initiates Clearance of Viable or Apoptotic Cells through Trans-Activation of LRP on the Phagocyte. Cell.

[13] Garg A.D., Leggatt G.R., Land W.G., Bianchi M.E., Vénéreau E., Ceriotti C. (2015). DAMPs from Cell Death to New Life. Article.

[14] Chen C., Xu P. (2023). Cellular Functions of CGAS-STING Signaling Cell Biology. Trends Cell Biol..

[15] Hopfner K.-P., Hornung V. (2020). Cell Intrinsic Recognition and Defence Systems against Foreign Genetic Material Encompass. Nat. Rev. Mol. Cell Biol..

[16] Samson N., Ablasser A. (2022). The CGAS–STING Pathway and Cancer. Nat. Cancer.

[17] de Oliveira Mann C.C., Orzalli M.H., King D.S., Kagan J.C., Lee A.S.Y., Kranzusch P.J. (2019). Modular Architecture of the STING C-Terminal Tail Allows Interferon and NF-ΚB Signaling Adaptation. Cell Rep..

[18] Huang L., Li L., Lemos H., Chandler P.R., Pacholczyk G., Baban B., Barber G.N., Hayakawa Y., McGaha T.L., Ravishankar B. (2013). Cutting Edge: DNA Sensing via the STING Adaptor in Myeloid Dendritic Cells Induces Potent Tolerogenic Responses. An Alternative Splicing Isoform of MITA Antagonizes MITA-Mediated Induction of Type I IFNs. J. Immunol..

[19] Larkin B., Ilyukha V., Sorokin M., Buzdin A., Vannier E., Poltorak A. (2017). Cutting Edge: Activation of STING in T Cells Induces Type I IFN Responses and Cell Death. J. Immunol..

[20] Kabelitz D., Lathia J., Nita-Lazar A., Hupp T.R., Goodlett D.R., Khan M.M., Allen Hamilton B., Urban-Wojciuk Z., Oyler B.L., Fåhraeus R. (2019). The Role of TLRs in Anti-Cancer Immunity and Tumor Rejection. Front. Immunol..

[21] Lu H., Yang Y., Gad E., Inatsuka C., Wenner C.A., Disis M.L., Standish L.J. (2011). TLR2 Agonist PSK Activates Human NK Cells and Enhances the Antitumor Effect of HER2-Targeted Monoclonal Antibody Therapy. Clin. Cancer Res..

[22] Ke M., Wang H., Zhang M., Tian Y., Wang Y., Li B., Yu J., Dou J., Xi T., Zhou C. (2014). The Anti-Lung Cancer Activity of SEP Is Mediated by the Activation and Cytotoxicity of NK Cells via TLR2/4 in Vivo. Biochem. Pharmacol..

[23] Deng Y., Yang J., Qian J., Liu R., Huang E., Wang Y., Luo F., Chu Y. (2019). TLR1/TLR2 Signaling Blocks the Suppression of Monocytic Myeloid-Derived Suppressor Cell by Promoting Its Differentiation into M1-Type Macrophage. Mol. Immunol..

[24] Wang Y., Su L., Morin M.D., Jones B.T., Mifune Y., Shi H., Wang K.-W., Zhan X., Liu A., Wang J. (2018). Adjuvant Effect of the Novel TLR1/TLR2 Agonist Diprovocim Synergizes with Anti-PD-L1 to Eliminate Melanoma in Mice. Proc. Natl. Acad. Sci. USA.

[25] Kiura K., Hasebe A., Saeki A., Segawa T., Okada F., Shamsul H.M., Ohtani M., Into T., Inoue N., Wakita M. (2011). In Vivo Anti- and pro-Tumour Activities of the TLR2 Ligand FSL-1. Immunobiology.

[26] Pandey S., Singh S., Anang V., Bhatt A.N., Natarajan K., Dwarakanath B.S. (2015). Pattern Recognition Receptors in Cancer Progression and Metastasis. Cancer Growth Metastasis.

[27] El-Kharashy G., Gowily A., Okda T., Houssen M. (2021). Association between Serum Soluble Toll-like Receptor 2 and 4 and the Risk of Breast Cancer. Mol. Clin. Oncol..

[28] Blazquez R., Chuang H.-N., Wenske B., Trigueros L., Wlochowitz D., Liguori R., Ferrazzi F., Regen T., Proescholdt M.A., Rohde V. (2022). ARTICLE Intralesional TLR4 Agonist Treatment Strengthens the Organ Defense against Colonizing Cancer Cells in the Brain. Oncogene.

[29] Di Lorenzo A., Bolli E., Ruiu R., Ferrauto G., Di Gregorio E., Avalle L., Savino A., Poggio P., Merighi I.F., Riccardo F. (2022). Toll-like Receptor 2 Promotes Breast Cancer Progression and Resistance to Chemotherapy. Oncoimmunology.

[30] De Angelis M.L., Francescangeli F., Zeuner A. (2019). Cancers Breast Cancer Stem Cells as Drivers of Tumor Chemoresistance, Dormancy and Relapse: New Challenges and Therapeutic Opportunities. Cancers.

[31] Conti L., Lanzardo S., Arigoni M., Antonazzo R., Radaelli E., Cantarella D., Calogero R.A., Cavallo F. (2013). The Noninflammatory Role of High Mobility Group Box 1/Toll-like Receptor 2 Axis in the Self-Renewal of Mammary Cancer Stem Cells. FASEB J. Res. Commun..

[32] Chen R., Kang R., Tang D. (2022). The Mechanism of HMGB1 Secretion and Release. Exp. Mol. Med..

[33] Sims G.P., Rowe D.C., Rietdijk S.T., Herbst R., Coyle A.J. (2010). HMGB1 and RAGE in Inflammation and Cancer. Annu. Rev. Immunol..

[34] Wang Z., Yang C., Li L., Jin X., Zhang Z., Zheng H., Pan J., Shi L., Jiang Z., Su K. (2020). Oncogenesis Tumor-Derived HMGB1 Induces CD62L Dim Neutrophil Polarization and Promotes Lung Metastasis in Triple-Negative Breast Cancer. Oncogenesis.

[35] Seclì L., Avalle L., Poggio P., Fragale G., Cannata C., Conti L., Iannucci A., Carr G., Rubinetto C., Miniscalco B. (2021). Targeting the Extracellular HSP90 Co-Chaperone Morgana Inhibits Cancer Cell Migration and Promotes Anticancer Immunity. Cancer Res..

[36] Kim S., Takahashi H., Lin W.-W., Descargues P., Grivennikov S., Kim Y., Luo J.-L., Karin M. (2009). LETTERS Carcinoma-Produced Factors Activate Myeloid Cells through TLR2 to Stimulate Metastasis. Nature.

[37] Di Lorenzo A., Bolli E., Tarone L., Cavallo F., Conti L. (2020). Toll-like Receptor 2 at the Crossroad between Cancer Cells, the Immune System, and the Microbiota. Int. J. Mol. Sci..

[38] Xie W., Wang Y., Huang Y., Yang H., Wang J., Hu Z. (2009). Toll-like Receptor 2 Mediates Invasion via Activating NF-ΚB in MDA-MB-231 Breast Cancer Cells. Biochem. Biophys. Res. Commun..

[39] Scheeren F.A., Kuo A.H., Van Weele L.J., Cai S., Glykofridis I., Sikandar S.S., Zabala M., Qian D., Lam J.S., Johnston D. (2014). A Cell-Intrinsic Role for TLR2-MYD88 in Intestinal and Breast Epithelia and Oncogenesis. Nat. Cell Biol..

[40] Schwitalla S., Fingerle A.A., Cammareri P., Nebelsiek T., Gö Ktuna S.I., Ziegler P.K., Canli O., Heijmans J., Huels D.J., Moreaux G. (2013). Intestinal Tumorigenesis Initiated by Dedifferentiation and Acquisition of Stem-Cell-like Properties. Cell.

[41] Tye H., Kennedy C.L., Najdovska M., Mcleod L., Mccormack W., Hughes N., Dev A., Sievert W., Ooi C.H., Ishikawa T.-O. (2012). Article STAT3-Driven Upregulation of TLR2 Promotes Gastric Tumorigenesis Independent of Tumor Inflammation. Cancer Cell.

[42] Chen X., Zhang Y., Fu Y. (2022). The Critical Role of Toll-like Receptor-Mediated Signaling in Cancer Immunotherapy. Med. Drug Discov..

[43] Parida S., Sharma D. (2019). The Power of Small Changes: Comprehensive Analyses of Microbial Dysbiosis in Breast Cancer. Biochim. Biophys. Acta Rev. Cancer.

[44] Huang B., Zhao J., Shen S., Li H., He K.-L., Shen G.-X., Mayer L., Unkeless J., Li D., Yuan Y. (2007). Listeria Monocytogenes Promotes Tumor Growth via Tumor Cell Toll-Like Receptor 2 Signaling. Cancer Res..

[45] Krzysiek-Maczka G., Targosz A., Szczyrk U., Strzalka M., Brzozowski T., Ptak-Belowska A. (2019). Involvement of Epithelial-Mesenchymal Transition-Inducing Transcription Factors in the Mechanism of Helicobacter Pylori-Induced Fibroblasts Activation. J. Physiol. Pharmacol..

[46] Parida S., Wu S., Siddharth S., Wang G., Muniraj N., Nagalingam A., Hum C., Mistriotis P., Hao H., Conover C. (2021). A Procarcinogenic Colon Microbe Promotes Breast Tumorigenesis and Metastatic Progression and Concomitantly Activates Notch and B-Catenin Axes. Cancer Discov..

[47] Kahraman Gürsoy U., Kantarci A., Enersen M., Guncu G.N., Bachrach G., Abed J., Maalouf N., Parhi L., Chaushu S., Mandelboim O. (2017). Tumor Targeting by Fusobacterium Nucleatum: A Pilot Study and Future Perspectives. Front. Cell. Infect. Microbiol..

[48] Jia Y.-P., Wang K., Zhang Z.-J., Tong Y.-N., Han D., Hu C.-Y., Li Q., Xiang Y., Mao X.-H., Tang B. (2017). TLR2/TLR4 Activation Induces Tregs and Suppresses Intestinal Inflammation Caused by Fusobacterium Nucleatum in vivo. PLoS ONE.

[49] Chiba A., Bawaneh A., Velazquez C., Clear K.Y.J., Wilson A.S., Howard-Mcnatt M., Levine E.A., Levi-Polyachenko N., Yates-Alston S.A., Diggle S.P. (2019). Neoadjuvant Chemotherapy Shifts Breast Tumor Microbiota Populations to Regulate Drug Responsiveness and the Development of Metastasis. Mol. Cancer Res..

[50] Montassier E., Gastinne T., Vangay P., Al-Ghalith G.A., Bruley Des Varannes S., Massart S., Moreau P., Potel G., De La Cochetière M.F., Batard E. (2015). Chemotherapy-Driven Dysbiosis in the Intestinal Microbiome. Aliment. Pharmacol. Ther..

[51] Bernardo G., Le Noci V., Ottaviano E., De Cecco L., Camisaschi C., Guglielmetti S., Di Modica M., Gargari G., Bianchi F., Indino S. (2023). Reduction of Staphylococcus Epidermidis in the Mammary Tumor Microbiota Induces Antitumor Immunity and Decreases Breast Cancer Aggressiveness. Cancer Lett..

[52] Sutmuller R.P.M., Den Brok M.H.M.G.M., Kramer M., Bennink E.J., Toonen L.W.J., Kullberg B.-J., Joosten L.A., Akira S., Netea M.G., Adema G.J. (2006). Toll-like Receptor 2 Controls Expansion and Function of Regulatory T Cells. J Clin Invest..

[53] Mcbride A., Konowich J., Salgame P. (2013). Host Defense and Recruitment of Foxp3 + T Regulatory Cells to the Lungs in Chronic Mycobacterium Tuberculosis Infection Requires Toll-like Receptor 2. PLoS Pathog..

[54] Ye L., Zhang Q., Cheng Y., Chen X., Wang G., Shi M., Zhang T., Cao Y., Pan H., Zhang L. (2018). Tumor-Derived Exosomal HMGB1 Fosters Hepatocellular Carcinoma Immune Evasion by Promoting TIM-1+ Regulatory B Cell Expansion. J. Immunother. Cancer.

[55] Chen Y.-Q., Li P.-C., Pan N., Gao R., Wen Z.-F., Zhang T.-Y., Huang F., Wu F.-Y., Ou X.-L., Zhang J.-P. (2019). Tumor-Released Autophagosomes Induces CD4 + T Cell-Mediated Immunosuppression via a TLR2-IL-6 Cascade. J. Immunother. Cancer.

[56] Niu X., Yin L., Yang X., Yang Y., Gu Y., Sun Y., Yang M., Wang Y., Zhang Q., Ji H. (2022). Serum Amyloid A 1 Induces Suppressive Neutrophils through the Toll-like Receptor 2–Mediated Signaling Pathway to Promote Progression of Breast Cancer. Cancer Sci..

[57] Andzinski L., Spanier J., Kasnitz N., Kröger A., Jin L., Brinkmann M.M., Kalinke U., Weiss S., Jablonska J., Lienenklaus S. (2016). Growing Tumors Induce a Local STING Dependent Type I IFN Response in Dendritic Cells. Int. J. Cancer.

[58] Sen T., Rodriguez B.L., Chen L., Della Corte C.M., Morikawa N., Fujimoto J., Cristea S., Nguyen T., Diao L., Li L. (2019). Targeting DNA Damage Response Promotes Antitumor Immunity through STING-Mediated T-Cell Activation in Small Cell Lung Cancer. Cancer Discov..

[59] Vargas-Rondón N., Villegas V.E., Rondón-Lagos M. (2018). The Role of Chromosomal Instability in Cancer and Therapeutic Responses. Cancers.

[60] Bakhoum S.F., Cantley L.C. (2018). The Multifaceted Role of Chromosomal Instability in Cancer and Its Microenvironment. Cell.

[61] Diamond M.S., Kinder M., Matsushita H., Mashayekhi M., Dunn G.P., Archambault J.M., Lee H., Arthur C.D., White J.M., Kalinke U. (2011). Type I Interferon Is Selectively Required by Dendritic Cells for Immune Rejection of Tumors. J. Exp. Med..

[62] Zhang L., Wei X., Wang Z., Liu P., Hou Y., Xu Y., Su H., Koci M.D., Yin H., Zhang C. (2023). NF-ΚB Activation Enhances STING Signaling by Altering Microtubule-Mediated STING Trafficking. Cell Rep..

[63] Kitai Y., Kawasaki T., Sueyoshi T., Kobiyama K., Ishii K.J., Zou J., Akira S., Matsuda T., Kawai T. (2017). DNA-Containing Exosomes Derived from Cancer Cells Treated with Topotecan Activate a STING-Dependent Pathway and Reinforce Antitumor Immunity. J. Immunol..

[64] Woo S.R., Fuertes M.B., Corrales L., Spranger S., Furdyna M.J., Leung M.Y.K., Duggan R., Wang Y., Barber G.N., Fitzgerald K.A. (2014). STING-Dependent Cytosolic DNA Sensing Mediates Innate Immune Recognition of Immunogenic Tumors. Immunity.

[65] Ritchie C., Cordova A.F., Hess G.T., Bassik M.C., Li L. (2019). SLC19A1 Is an Importer of the Immunotransmitter CGAMP. Mol. Cell.

[66] Maltbaek J.H., Cambier S., Snyder J.M., Stetson D.B. (2022). ABCC1 Transporter Exports the Immunostimulatory Cyclic Dinucleotide CGAMP. Immunity.

[67] Marcus A., Mao A.J., Lensink-Vasan M., Wang L.A., Vance R.E., Raulet D.H. (2018). Tumor-Derived CGAMP Triggers a STING-Mediated Interferon Response in Non-Tumor Cells to Activate the NK Cell Response. Immunity.

[68] Lu L., Yang C., Zhou X., Wu L., Hong X., Li W., Wang X., Yang Y., Cao D., Zhang A. (2023). STING Signaling Promotes NK Cell Antitumor Immunity and Maintains a Reservoir of TCF-1+ NK Cells. Cell Rep..

[69] Cordova A.F., Ritchie C., Böhnert V., Li L. (2021). Human SLC46A2 Is the Dominant CGAMP Importer in Extracellular CGAMP-Sensing Macrophages and Monocytes. ACS Cent. Sci..

[70] Wang Q., Bergholz J.S., Ding L., Lin Z., Kabraji S.K., Hughes M.E., He X., Xie S., Jiang T., Wang W. (2022). STING Agonism Reprograms Tumor-Associated Macrophages and Overcomes Resistance to PARP Inhibition in BRCA1-Deficient Models of Breast Cancer. Nat. Commun..

[71] An X., Zhu Y., Zheng T., Wang G., Zhang M., Li J., Ji H., Li S., Yang S., Xu D. (2019). An Analysis of the Expression and Association with Immune Cell Infiltration of the CGAS/STING Pathway in Pan-Cancer. Mol. Ther. Nucleic Acids.

[72] Xia T., Konno H., Ahn J., Barber G.N. (2016). Deregulation of STING Signaling in Colorectal Carcinoma Constrains DNA Damage Responses and Correlates with Tumorigenesis. Cell Rep..

[73] Falahat R., Berglund A., Putney R.M., Perez-Villarroel P., Aoyama S., Pilon-Thomas S., Barber G.N., Mulé J.J. (2021). Epigenetic Reprogramming of Tumor Cell-Intrinsic STING Function Sculpts Antigenicity and T Cell Recognition of Melanoma. Proc. Natl. Acad. Sci. USA.

[74] Zheng H., Wu L., Xiao Q., Meng X., Hafiz A., Yan Q., Lu R., Cao J. (2023). Epigenetically Suppressed Tumor Cell Intrinsic STING Promotes Tumor Immune Escape. Biomed. Pharmacother..

[75] Bakhoum S.F., Ngo B., Laughney A.M., Cavallo J.A., Murphy C.J., Ly P., Shah P., Sriram R.K., Watkins T.B.K., Taunk N.K. (2018). Chromosomal Instability Drives Metastasis through a Cytosolic DNA Response. Nature.

[76] Hanahan D., Weinberg R.A. (2011). Hallmarks of Cancer: The next Generation. Cell.

[77] Voutsadakis I.A. (2021). The Landscape of Chromosome Instability in Breast Cancers and Associations with the Tumor Mutation Burden: An Analysis of Data from TCGA. Cancer Investig..

[78] Hong C., Schubert M., Tijhuis A.E., Requesens M., Roorda M., van den Brink A., Ruiz L.A., Bakker P.L., van der Sluis T., Pieters W. (2022). CGAS–STING Drives the IL-6-Dependent Survival of Chromosomally Instable Cancers. Nature.

[79] Vasiyani H., Mane M., Rana K., Shinde A., Roy M., Singh J., Gohel D., Currim F., Srivastava R., Singh R. (2022). DNA Damage Induces STING Mediated IL-6-STAT3 Survival Pathway in Triple-Negative Breast Cancer Cells and Decreased Survival of Breast Cancer Patients. Apoptosis.

[80] Cheon H., Holvey-Bates E.G., Mcgrail D.J., Stark G.R., Diaz L.A., Hertzog P.J. (2021). PD-L1 Sustains Chronic, Cancer Cell-Intrinsic Responses to Type I Interferon, Enhancing Resistance to DNA Damage. Proc. Natl. Acad. Sci. USA.

[81] Gaston J., Cheradame L., Yvonnet V., Deas O., Poupon M.-F., Judde J.-G., Cairo S., Goffin V. (2016). Intracellular STING Inactivation Sensitizes Breast Cancer Cells to Genotoxic Agents. Oncotarget.

[82] Wang J., Yi S., Zhou J., Zhang Y., Guo F. (2016). The NF-B Subunit RelB Regulates the Migration and Invasion Abilities and the Radio-Sensitivity of Prostate Cancer Cells. Int. J. Oncol..

[83] Ghosh M., Saha S., Bettke J., Nagar R., Parrales A., Iwakuma T., van der Velden A.W.M., Martinez L.A. (2021). Mutant P53 Suppresses Innate Immune Signaling to Promote Tumorigenesis. Cancer Cell.

[84] Khan S., Bijker M.S., Weterings J.J., Tanke H.J., Adema G.J., Van Hall T., Drijfhout J.W., Melief C.J.M., Overkleeft H.S., Van Der Marel G.A. (2007). Distinct Uptake Mechanisms but Similar Intracellular Processing of Two Different Toll-like Receptor Ligand-Peptide Conjugates in Dendritic Cells. J. Biol. Chem..

[85] Zom G.G., Khan S., Britten C.M., Sommandas V., Camps M.G.M., Loof N.M., Budden C.F., Meeuwenoord N.J., Filippov D.V., van der Marel G.A. (2014). Efficient Induction of Antitumor Immunity by Synthetic Toll-like Receptor Ligand-Peptide Conjugates. Cancer Immunol. Res..

[86] Abdel-Aal A.B.M., Lakshminarayanan V., Thompson P., Supekar N., Bradley J.M., Wolfert M.A., Cohen P.A., Gendler S.J., Boons G.J. (2014). Immune and Anticancer Responses Elicited by Fully Synthetic Aberrantly Glycosylated MUC1 Tripartite Vaccines Modified by a TLR2 or TLR9 Agonist. ChemBioChem.

[87] Shi W., Tong Z., Chen S., Qiu Q., Zhou J., Qian H. (2023). Development of Novel Self-Assembled Vaccines Based on Tumour-Specific Antigenic Peptide and TLR2 Agonist for Effective Breast Cancer Immunotherapy via Activating CD8+ T Cells and Enhancing Their Function. Immunology.

[88] Liu B., Huang J., Xiao J., Xu W., Zhang H., Yuan Y., Yin Y., Zhang X. (2023). RESEARCH Open Access the Streptococcus Virulence Protein PepO Triggers Anti-Tumor Immune Responses by Reprograming Tumor-Associated Macrophages in a Mouse Triple Negative Breast Cancer Model. Cell Biosci..

[89] Lu H., Yang Y., Gad E., Wenner C.A., Chang A., Larson E.R., Dang Y., Martzen M., Standish L.J., Disis M.L. (2011). Cancer Therapy: Preclinical Polysaccharide Krestin Is a Novel TLR2 Agonist That Mediates Inhibition of Tumor Growth via Stimulation of CD8 T Cells and NK Cells. Clin. Cancer Res..

[90] Corrales L., Glickman L.H., Dubensky T.W., Gajewski T.F. (2015). Direct Activation of STING in the Tumor Microenvironment Leads to Potent and Systemic Tumor Regression and Immunity. Cell Rep..

[91] Baird J.R., Friedman D., Cottam B., Dubensky T.W., Kanne D.B., Bambina S., Bahjat K., Crittenden M.R., Gough M.J. (2016). Microenvironment and Immunology Radiotherapy Combined with Novel STING-Targeting Oligonucleotides Results in Regression of Established Tumors. Cancer Res.

[92] Lu X., Wang X., Cheng H., Wang X., Liu C., Tan X. (2023). Anti-Triple-Negative Breast Cancer Metastasis Efficacy and Molecular Mechanism of the STING Agonist for Innate Immune Pathway. Ann. Med..

[93] Chandra D., Quispe-Tintaya W., Jahangir A., Asafu-Adjei D., Ramos I., Sintim H.O., Zhou J., Hayakawa Y., Karaolis D.K.R., Gravekamp C. (2014). STING Ligand C-Di-GMP Improves Cancer Vaccination against Metastatic Breast Cancer. Cancer Immunol. Res..

[94] Yin M., Hu J., Yuan Z., Luo G., Yao J., Wang R., Liu D., Cao B., Wu W., Hu Z. (2022). STING Agonist Enhances the Efficacy of Programmed Death-Ligand 1 Monoclonal Antibody in Breast Cancer Immunotherapy by Activating the Interferon-β Signalling Pathway. Cell Cycle.

[95] Watkins-Schulz R., Batty C.J., Stiepel R.T., Schmidt M.E., Sandor A.M., Chou W.C., Ainslie K.M., Bachelder E.M., Ting J.P.Y. (2022). Microparticle Delivery of a STING Agonist Enables Indirect Activation of NK Cells by Antigen-Presenting Cells. Mol. Pharm..

[96] Conlon J., Burdette D.L., Sharma S., Bhat N., Thompson M., Jiang Z., Rathinam V.A.K., Monks B., Jin T., Xiao T.S. (2013). Mouse, but Not Human STING, Binds and Signals in Response to the Vascular Disrupting Agent 5,6-Dimethylxanthenone-4-Acetic Acid. J. Immunol..

[97] Gao P., Ascano M., Zillinger T., Wang W., Dai P., Serganov A.A., Gaffney B.L., Shuman S., Jones R.A., Deng L. (2013). Structure-Function Analysis of STING Activation by c[G(2′,5′)pA(3′,5′)p] and Targeting by Antiviral DMXAA. Cell.

[98] Hines J.B., Kacew A.J., Sweis R.F. (2023). The Development of STING Agonists and Emerging Results as a Cancer Immunotherapy. Curr. Oncol. Rep..

[99] Lin J.-S., Huang J.-H., Hung L.-Y., Wu S.-Y., Wu-Hsieh B.A. (2010). Distinct Roles of Complement Receptor 3, Dectin-1, and Sialic Acids in Murine Macrophage Interaction with Histoplasma Yeast. J. Leukoc. Biol..

[100] Garcia-Manero G., Jabbour E.J., Konopleva M.Y., Daver N.G., Borthakur G., DiNardo C.D., Bose P., Patel P., Komrokji R.S., Shastri A. (2018). A Clinical Study of Tomaralimab (OPN-305), a Toll-like Receptor 2 (TLR-2) Antibody, in Heavily Pre-Treated Transfusion Dependent Patients with Lower Risk Myelodysplastic Syndromes (MDS) That Have Received and Failed on Prior Hypomethylating Agent (HMA) Therapy. Blood.

[101] Zhang W., Liu W., Hu X. (2023). Robinin Inhibits Pancreatic Cancer Cell Proliferation, EMT and Inflammation via Regulating TLR2-PI3k-AKT Signaling Pathway. Cancer Cell Int..

[102] Farnebo L., Shahangian A., Lee Y., Shin J.H., Scheeren F.A., Sunwoo J.B., Farnebo L., Shahangian A., Lee Y., Shin J.H. (2015). Targeting Toll-like Receptor 2 Inhibits Growth of Head and Neck Squamous Cell Carcinoma. Oncotarget.

